# Relationship between the dual platelet‐inhibited ROTEM® Sigma FIBTEM assay and Clauss fibrinogen during postpartum haemorrhage

**DOI:** 10.1111/anae.16455

**Published:** 2024-10-25

**Authors:** Sarah F. Bell, Hazel Taylor, Philip Pallmann, Peter Collins, the OBS Cymru collaborators (online Supporting Information Appendix S1)

**Affiliations:** ^1^ Cardiff and Vale University Health Board Cardiff UK; ^2^ Cardiff University Cardiff UK

Fibrinogen is essential for haemostasis and can fall to critically low levels in acute haemorrhage [1]. The long turnaround time for laboratory Clauss fibrinogen has led to interest in point‐of‐care viscoelastic haemostatic assays to identify hypofibrinogenemia. The ROTEM® Delta and Sigma devices (Werfen, Warrington, UK) offer the FIBTEM assay to assess fibrinogen contribution to clot strength in whole blood. FIBTEM A5, the amplitude 5 min after the clotting time, is used as a surrogate for the Clauss fibrinogen in management algorithms [2, 3]. The original FIBTEM assay used Cytochalasin D to inhibit platelets although inhibition was found to be partially influenced by the platelet count [4]. Tirofiban, a glycoprotein 2b/3a receptor antagonist, was added to reduce the influence of platelets and the dual platelet‐inhibited assay received regulatory approval in 2022 [5].

Guidelines recommend that fibrinogen levels should be maintained > 2 g.l^‐1^ [6, 7] in obstetric haemorrhage. Since 2017, management of postpartum haemorrhage in Wales has followed the OBS Cymru ROTEM® algorithm [3] with a FIBTEM A5 > 11 mm corresponding to a Clauss fibrinogen of approximately 2 g.l^‐1^. In April 2023, Sigma cartridges with the dual platelet‐inhibited FIBTEM assay were distributed in the UK. Clinicians at our institution became aware of this change in July 2024 following anecdotal observations of an altered relationship between FIBTEM A5 and Clauss fibrinogen, and discussions with the manufacturer.

Following local service evaluation registration, anonymised data were collected retrospectively from five obstetric units in Wales using the dual platelet‐inhibited FIBTEM assay. In total, 212 paired FIBTEM and Clauss fibrinogen results were available for analysis with some patients having more than one sample during a single postpartum haemorrhage episode. Four samples from a patient with severe liver impairment were excluded. The utility of the dual platelet‐inhibited FIBTEM A5 to distinguish Clauss fibrinogen ≤ 2 g.l^‐1^ was analysed. Fibrinogen ≤ 2 g.l^‐1^ is uncommon during postpartum haemorrhage and to obtain sufficient data around this level, purposive data collection was necessary (Fig. 1). Comparison was made with data from a previous study which used single platelet‐inhibited Sigma FIBTEM assays [1].

**Figure 1:**
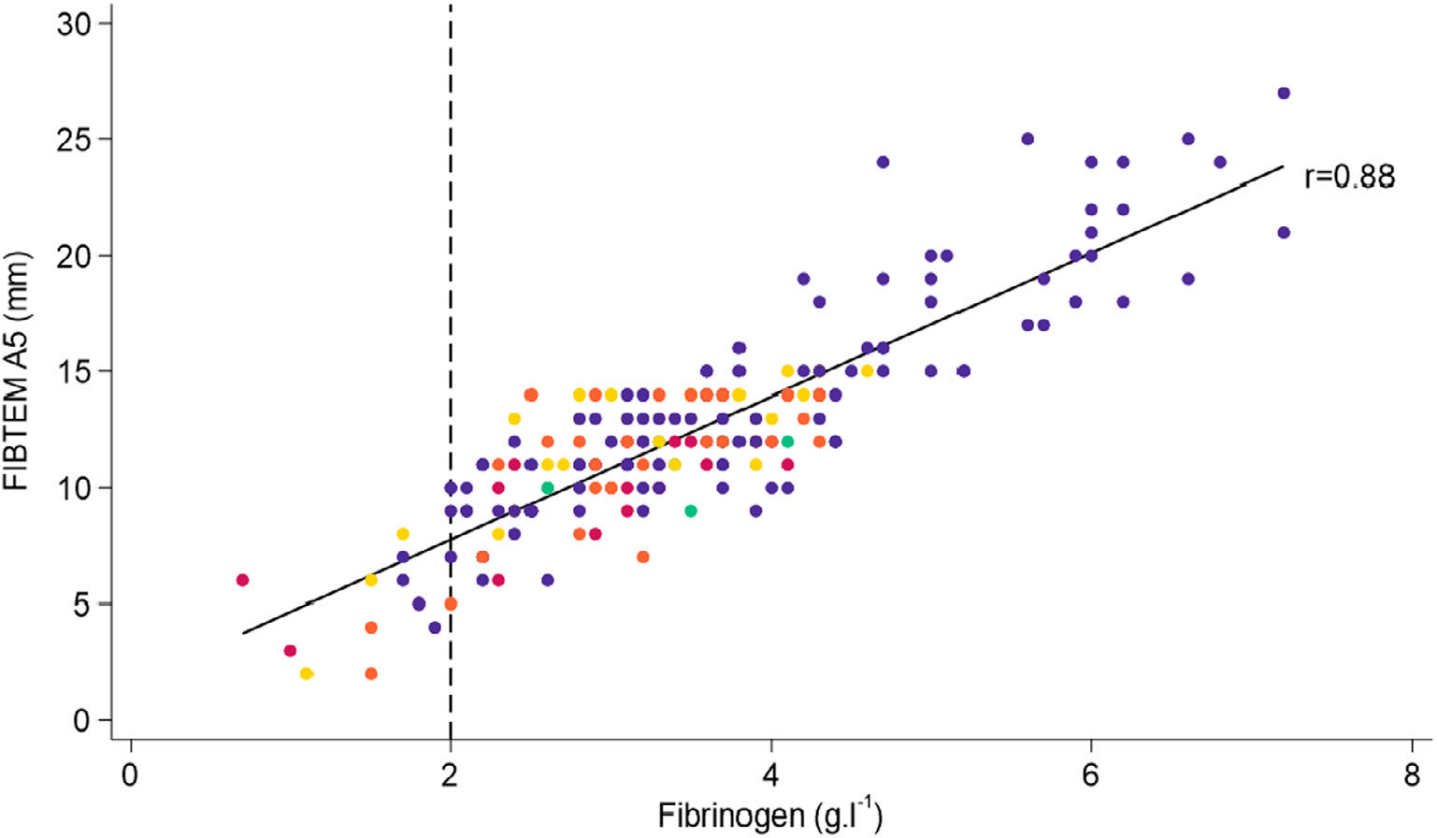
Linear correlation between Clauss fibrinogen and ROTEM® Sigma FIBTEM A5 dual platelet‐inhibited assay in obstetric haemorrhage. The regression line Y = 1.58 + 3.09X gives an estimate of the mean FIBTEM A5 for a given value of Class fibrinogen. Site 1 (blue dots) provided 115 paired samples (54 consecutive paired samples followed by purposive sampling of FIBTEM A5 ≤ 15 mm for the next 22 cases, then 39 cases for A5 ≤ 12 mm). Site 2 (green dots) provided seven paired samples with FIBTEM A5 ≤ 12 mm. Sites 3 (yellow dots), 4 (red dots) and 5 (orange dots) contributed 32, 21 and 33 paired samples with FIBTEM A5 ≤ 15 mm, respectively. In the case of multiple paired samples occurring on a single point, only one colour is shown. The platelet count was available in 200/208 samples with a median (IQR [range]) of 192 × 10^9^ l^‐1^ (147–238 [22–399]) and 6.5% (13/200) of results had a platelet count < 75 × 10^9^ l^‐1^. There was a weak correlation between FIBTEM A5 and platelet count (r = 0.24, p < 0.005).

There was a stronger linear correlation between FIBTEM A5 and Clauss fibrinogen (r = 0.88) (Fig. 1) in the dual platelet‐inhibited FIBTEM assay compared with data from a single platelet‐inhibited assay (r = 0.63) [8]. With the dual platelet‐inhibited assay, FIBTEM A5 of 11 mm (as used in the algorithm with the single platelet‐inhibited assay [3]) corresponded to a Clauss fibrinogen of 3.05 g.l^‐1^, while FIBTEM A5 of 7.8 mm corresponded to a Clauss fibrinogen of 2 g.l^‐1^. The area under the receiver operating characteristic curve for FIBTEM A5 to detect fibrinogen ≤ 2 g.l^‐1^, and sensitivity and specificity of FIBTEM A5 at different intervention points were compared between single and dual platelet‐inhibited assays (Table 1). With the dual platelet‐inhibited assay, a FIBTEM A5 ≤ 11 mm identified all patients with fibrinogen ≤ 2 g.l^‐1^, however of the 191/208 cases with fibrinogen > 2 g.l^‐1^, 71 had FIBTEM ≤ 11 mm and may have been inappropriately administered fibrinogen replacement therapy. With the dual platelet‐inhibited assay, a threshold of FIBTEM A5 ≤ 8 mm showed near identical positive and negative predictive values to the intervention point of ≤ 11 mm with the single platelet‐inhibited FIBTEM assay (Table 1). The OBS Cymru algorithm has been updated accordingly (online Supporting Information Figure S1).

**Table 1:** Area under the receiver operating characteristic curve (AUROC) for ROTEM® Sigma FIBTEM A5 to detect fibrinogen ≤ 2 g.l^‐1^.

FIBTEM assay type	AUROC (95%CI)	Threshold; mm	Sensitivity (95%CI)	Specificity (95%CI)	Positive predictive value (95%CI)	Negative predictive value (95%CI)	Youden index
Single‐platelet inhibition n = 552*	0.96 (0.94–0.98)	≤ 12	0.79 (0.61–0.91)	0.92 (0.89–0.94)	0.38 (0.27–0.51)	0.99 (0.97–0.99)	0.71
		≤ 11	0.76 (0.58–0.89)	0.96 (0.94–0.98)	0.57 (0.41–0.72)	0.98 (0.97–0.99)	0.72
		≤ 10	0.64 (0.45–0.80)	0.97 (0.96–0.99)	0.62 (0.44–0.78)	0.98 (0.96–0.99)	0.61
Dual platelet inhibition n = 208	0.97 (0.93–0.99)	≤ 11	1.00 (0.81–1.00)	0.63 (0.56,0.70)	0.19 (0.12–0.29)	1.00 (0.97–1.00)	0.63
		≤ 10	1.00 (0.81–1.00)	0.78 (0.71–0.83)	0.28 (0.18–0.41)	1.00 (0.98–1.00)	0.78
		≤ 9	0.88 (0.64–0.99)	0.87 (0.82–0.92)	0.38 (0.23–0.55)	0.99 (0.96–1.00)	0.75
		≤ 8	0.82 (0.57–0.96)	0.94 (0.90–0.97)	0.56 (0.35–0.76)	0.98 (0.95–1.00)	0.76
		≤ 7	0.76 (0.50–0.93)	0.96 (0.93–0.99)	0.65 (0.41–0.85)	0.98 (0.95–0.99)	0.72

* Data for the 552 cases with single platelet inhibition have been published previously [1].

The correlation between Clauss fibrinogen and FIBTEM A5 was stronger with the dual platelet‐inhibited assay when compared with the single platelet‐inhibited assay. We hypothesise that the enhanced platelet inhibition makes the FIBTEM assay more dependent on fibrinogen and hence a more useful surrogate marker. The change from a single to a dual platelet‐inhibited FIBTEM assay could not have been detected by internal quality control or external quality assurance because these use plasma‐based reagents, rather than whole blood. The difference in platelet inhibition was not detected because platelets are not present in the plasma‐based reagents. This emphasises the importance of pairing laboratory and point‐of‐care coagulation tests to monitor device performance. The manufacturer previously compared the clinical performance of the ROTEM® Sigma FIBTEM assay (with dual platelet inhibition) and the ROTEM Delta FIBTEM assay (with single platelet inhibition) in patients undergoing cardiac and liver surgery and found a mean bias of ‐1.5 to ‐1.8 mm for FIBTEM A5 of 12 mm [5]. The change in the Sigma FIBTEM assay may also have implications for sites that use the formula ‘EXTEM amplitude minus FIBTEM amplitude’ to guide platelet transfusion, with the potential for under transfusion. These considerations do not apply to the Delta FIBTEM because the assay has not changed. Further validation is urgently required to assess the impact of the dual platelet‐inhibited Sigma FIBTEM assay in other clinical settings. We highlight the importance of communicating all updates to point‐of‐care devices and reagents to end users so that the impact in different settings can be fully evaluated.

## Supporting information


**Figure S1.** Revised OBS Cymru ROTEM® Sigma interpretation algorithm based on an updated intervention point of ≤ 8 mm which has been adopted in Wales.

**Appendix S1.** OBS Cymru collaborators.
