# Supplementary figures and images for: Relationship between the dual platelet‐inhibited ROTEM® Sigma FIBTEM assay and Clauss fibrinogen during postpartum haemorrhage
Source: Anaesthesia. 2024 Oct 25;80(1):104–6. doi: 10.1111/anae.16455 (PMC11617128; doi:10.1111/anae.16455)

Figure S1

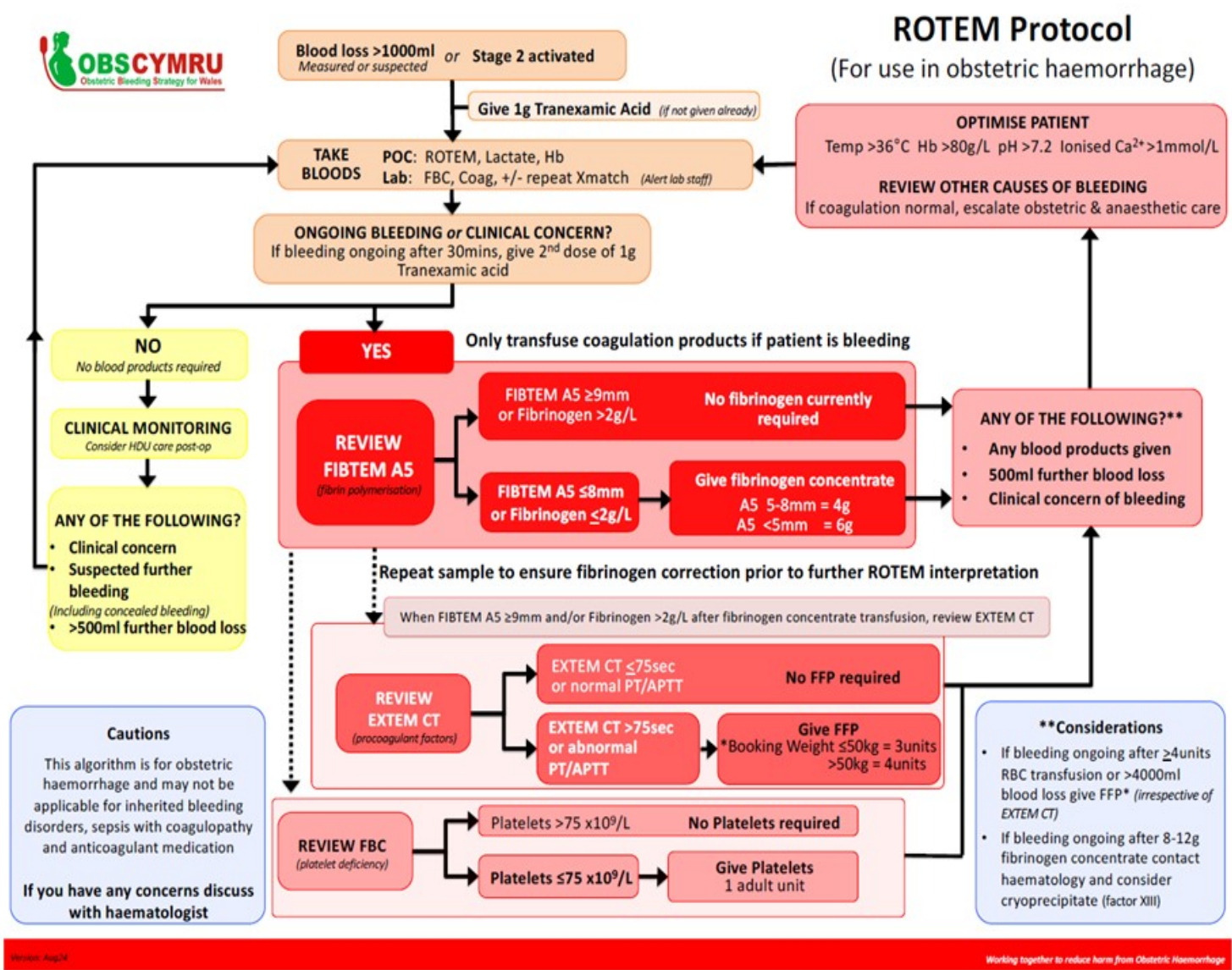

Supplement: Supplementary file 1 — Figure S1. Revised OBS Cymru ROTEM® Sigma interpretation algorithm based on an updated intervention point of ≤ 8 mm which has been adopted in Wales. [file ANAE-80-104-s001.pdf]
