# Supplementary material for: Relationship between the dual platelet‐inhibited ROTEM® Sigma FIBTEM assay and Clauss fibrinogen during postpartum haemorrhage
Source: Anaesthesia. 2024 Oct 25;80(1):104–6. doi: 10.1111/anae.16455 (PMC11617128; doi:10.1111/anae.16455)
Supplement: Supplementary file 2 — Appendix S1. OBS Cymru collaborators. [file ANAE-80-104-s002.docx]

**Online Supporting Information Appendix S1**

**OBS Cymru collaborators**

Peter V. Jenkins^8^, Rachel E. Collis^1^, Nicholas Preston^1^, Priscilla Chan^7^, Lucy de Lloyd^1^, Nicholas Gill^1^, Amrit Dhadda^1^, Nigel Jenkins^4^, Daniel Brunyseels^5^, Ingrid Volikas^6^, Xenia David^6^, James Tozer^7^, James Cutlan^7^, Helen Cordy^9^, Ruth Jones^10^.

1 Department of Anaesthetics, Cardiff and Vale University Health Board, Cardiff, UK.

2 Centre for Trials Research, Cardiff University, Cardiff, UK.

3 Institute of Infection and Immunity, School of Medicine, Cardiff University, Cardiff, UK.

4 Department of Anaesthetics, Swansea Bay University Health Board, Swansea, UK.

5 Department of Anaesthetics, Cwm Taf University Health Board, Merthyr Tydfil, UK.

6 Department of Anaesthetics, Betsi Cadwaladr University Health Board, Rhyl, UK.

7 Department of Anaesthetics, Aneurin Bevan University Health Board, Newport, UK.

8 Department of Haemostasis and Thrombosis, Cardiff and Vale University Health Board, Cardiff, UK.

9 Point of Care Testing Department, Cardiff and Vale University Health Board, Cardiff, UK.

10 Point of Care Testing Department, Swansea Bay University Health Board, Swansea, UK.
